# Phytochemical, Ethnobotanical, Pharmacological, and Toxicological Profile of *Globularia alypum* L.: A Comprehensive Review

**DOI:** 10.3390/plants14233641

**Published:** 2025-11-29

**Authors:** Fadoua Asraoui, Mariem Ben-Said, Nabila El Mansouri, Imane Bakkali, El Hassan Sakar, Mohamed Bakha, Noemí Ortiz-Liébana, Francesco Cacciola, Adnane Louajri, Miguel Palma Lovillo, Jamal Brigui, Fouad El Mansouri

**Affiliations:** 1 Laboratory of Biology, Ecology, and Health, Faculty of Sciences, Abdelmalek Essaadi University, Tetouan 93000, Morocco; f.asraoui@uae.ac.ma (F.A.); e.sakar@uae.ac.ma (E.H.S.); alouajri@uae.ac.ma (A.L.); 2 Laboratory of Chemical Engineering and Resources Valorization (LGCVR), Faculty of Sciences and Techniques, University Abdelmalek Essaadi, B. P. 416, Tangier 90010, Moroccoj.brigui@uae.ac.ma (J.B.); f.mansouri@uae.ac.ma (F.E.M.); 3 Unit of Plant Biotechnology and Sustainable Development of Natural Resources “B2DRN”, Poly-Disciplinary Faculty of Beni Mellal, Sultan Moulay Slimane University, Mghila, P.O. Box 592, Beni Mellal 23000, Morocco; m.bakha@usms.ma; 4 Institute of Environment, Natural Resources and Biodiversity, University of León, 24009 León, Spain; nortl@unileon.es; 5 Messina Institute of Technology c/o Department of Chemical, Biological, Pharmaceutical and Environmental Sciences, Former Veterinary School, University of Messina, Viale G. Palatucci snc, 98168 Messina, Italy; 6 Department of Analytical Chemistry, Faculty of Sciences, Instituto de Investigación Vitivinícola y Agroalimentaria (IVAGRO), University of Cadiz, Campus del Rio San Pedro, 11510 Cadiz, Spain; miguel.palma@uca.es

**Keywords:** *Globularia. alypum* L., traditional uses, herbal medicine, phytochemistry, bioactive compounds, pharmacology

## Abstract

*Globularia alypum* L. (Plantaginaceae) is widespread in the Mediterranean region and traditionally used against diabetes, digestive disorders, infections, and skin problems. This review summarizes its botanical features, ethnobotanical uses, phytochemistry, pharmacological effects, and toxicological profile. Relevant studies published between 1991 and 2024 were retrieved from Web of Science, Scopus, PubMed, and other relevant databases using targeted keywords in English and French. Extracts of *G. alypum* have shown significant antidiabetic, antioxidant, anticancer, antibacterial, anti-inflammatory, anticoagulant, nephroprotective, and wound-healing activities in vitro and in vivo, which were largely attributed to its diverse secondary metabolites such as phenolics, flavonoids, and iridoids. Toxicological studies indicate generally low risk at tested doses. However, further research is needed to elucidate the molecular mechanisms underlying these activities, validate its efficacy through clinical trials, and evaluate long-term safety, thereby bridging traditional knowledge with modern pharmacological evidence.

## 1. Introduction

Since early times, phytotherapy has incited scientific interest for the wide range of natural substances extracted from medicinal plants [1,2,3]. It has been proved that many of these plants do have a strong potential as alternative medications against various infectious diseases to be advantageous for food preservation and in the cosmetic industries [1,4,5].

In the last two decades, a focus on the link between plant phytoconstituents and human health has taken considerable place. Phytochemical substances such as vitamins, polyphenols, flavonoids, and phytosterols are acknowledged to prevent innumerable human diseases [6,7].

Thanks to its rich and diverse flora, the traditional pharmacopoeia in Morocco provides a wide arsenal of plant remedies [8]. Moroccan people have a long therapeutic tradition related to a rich traditional know-how of plant medications, which forms a part of their civilization [2,8]. There are about 800 taxa potentially used as aromatic and medicinal plants in Morocco [2,9].

**Figure 1:**
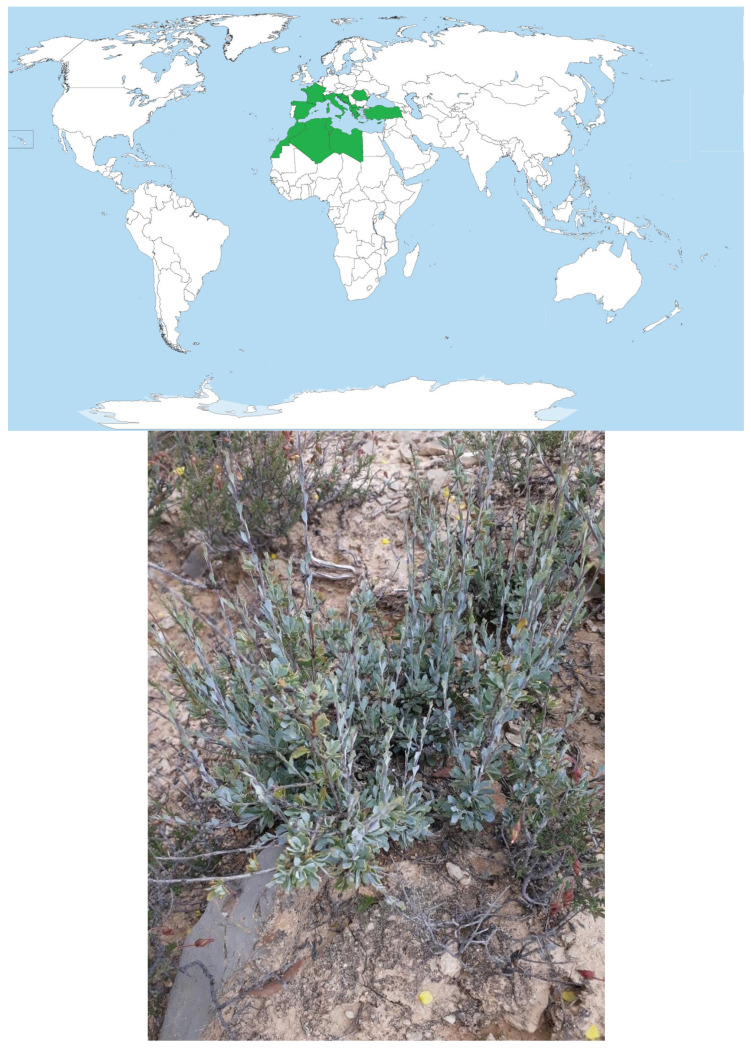
Distribution area of *Globularia alypum* in the world and illustration of the species collected in Taza region, northern Morocco.

The family Plantaginaceae (Globulariaceae) is formed by shrubs or sub-shrubs with zygomorphic flowers [10]. In Morocco, there is one genus and three species, namely *G. naini* Batt. and *G. liouvillei* Jahand. & Maire, both endemic [10,11], whereas the third one, *G. alypum* L., is common in shrublands of Mediterranean climate regions [10,12,13] and is native to Morocco, Algeria, Tunisia, Libya, Turkey, Serbia, Montenegro, Bosnia and Herzegovina, Croatia, North Macedonia, Slovenia, Albania, Baleares, Corse, East Aegean, Greece, Italy, Kriti, Sardinia, Sicilia, France, and Spain (Figure 1).

*Globularia alypum* L. (Alypo Globe Daisy, *G. alypum* hereafter) is a wild perennial evergreen shrub [10,13]. It has erected ligneous stems, its leaves are spatulate, lanceolate, or slightly tridentate at the apex; the flowers are purplish blue with a very short upper lip and perceptible and imbricate bracts [10,14]. It is a seeder and resprouting species [14]. In Morocco, *G. alypum* is found in *Tetraclinis articulata* (Vahl) Masters and *Pinus halepensis* Miller forest stands, as well as in scrublands and matorrals, in Infra- and Meso-Mediterranean vegetation belts from the plains up to altitudes around 1800 m, under the hot and fresh variants of the semi-arid and subhumid bioclimates, and it often grows on calcareous and superficial soils [10].

Known locally as” Taselgha” or” Ain Larneb” (i.e., rabbit’s eye), *G. alypum* constitutes one of the most remarkable herbs not only in Moroccan [8,15,16] but also in Arab folk medicine such as Algerian [6,17,18] and Tunisian [19,20]. Ethnobotanical surveys revealed that the treatment of numerous diseases using this medicinal plant is common across different Moroccan regions, including oriental [21,22] north center (Fez–Boulemane: [8]; Meknès-Tafilalet: [23]; Al Haouz-Rhamna: [24]; Sefrou: [25]; central Middle Atlas: [26]; southern (Tafilalet: [27]; Souss Massa Draa: [5]; Taroudant: [28]; Sahara (Tan-Tan: [29], and more recently in northern region (Taza: [3]). In traditional medicine, ethnobotanical surveys reported that *G. alypum* is widely used, either as decoction or infusion, against various health disorders and diseases. Indeed, it is used as cholagogue, hypoglycemic, laxative, purgative, stomachic, stimulant, depurative, diuretic, and sudorific agent, as well as in the treatment of diabetes, anemia, boils, intermittent fever, constipation, arthritis, skin diseases especially eczema, gout, rheumatism, intermittent fever, typhoid, renal, and cardiovascular disorders [6,8,13,20,30,31,32,33,34,35,36,37].

Different phytochemical analyses indicated that *G. alypum* encompasses numerous classes of bioactive compounds such as phenolic compounds [3,4,7,13,14,20,37,38,39,40,41,42,43,44], phenylethanoids [13,35,37,45,46], phenylethyl glycosides [47,48], flavonoids [3,4,5,7,13,14,19,20,35,38,39,41,42,43,45,48,49], terpenoids [4], anthocyanins [20,38,43,50], iridoids [3,35,37,45,46,48,51], tannins [7,19,43], coumarins [19], sterols [19], phytosterols [7], and secoiridoids [35], as well as volatile compounds [3]. In addition, the important nutritional value of the species is noticeable thanks to the presence of four vitamins (i.e., D, K, E, and A), several minerals (mainly Ca, Na, K, Fe, Mn, Zn, and Mg), five sugars (i.e., arabinose, fructose, glucose, sucrose, and maltose), and high protein contents [7].

Numerous in vitro and in vivo investigations using different parts of the plant (i.e., leaves, flowers, stems, and branches), have proved that *G. alypum* extracts, and to a lesser extent essential oils, had several biological effects such as antioxidant [1,3,5,6,13,14,19,20,34,38,39,40,41,42,45,52,53], antihyperglycemic [4,8,18,34,41,42,51,54], antibacterial [1,40,41,43,55], anti-inflammatory [40,46,56,57], burn wound healing [40], antiproliferative and cytotoxic [20], antihypertriglyceridemic and antihypercholesterolemic [4,51,58], anti-ulcer [59], antifungal [1,3,41], antiviral [60], vasodilatory [61], antileukemic [62], good protection against lipid peroxidation [4,20], spermatogenesis activation [63], as well as inhibition of radical-induced red blood cell hemolysis inhibition [6,42]. Furthermore, the plant has been reported to be active against neoplastic cell culture [37,64], and constipation [65]. It is also reported to decrease plasma lipids, creatinine, urea, transaminases activities [34], and nifuroxazide genotoxicity in *Escherichia coli* [19].

Most of its biological activities have been linked to the different bioactives formerly described in *G. alypum*, such as phenolic compounds and iridoids [13,14,19,40,42,43,45,66,67]. Compared to other medicinal plants, *G. alypum* was found to be among the species with the highest total phenolic levels [5,6,7], and thus the highest antioxidant activity [1,7,39].

Recently, several review papers have explored the properties of medicinal plants such as *Thymus* satureioides Coss. [68], *Matricaria chamomilla* L. [69], *Centaurium erythraea* Rafn. [70], *Daphne gnidium* L. [71], *Chenopodium album* L. [72], *Bulbophyllum* spp. [73], *Chamaerops humilis* [74], and *Crocus sativus* [75]. Despite the abundant literature reporting the ethnomedicinal uses and pharmacological properties of *G. alypum*, to our knowledge, there is no systematic review on the use of *G. alypum* as a potential source of natural substances having diverse biological activities against several diseases and health disorders, which could drive future studies for this species.

The present narrative review aims to critically summarize all studies on ethnomedicinal uses, phytochemistry, and pharmacological activities of *G. alypum*, as well as to discuss future challenges and opportunities. Indeed, this review is not only important because it resumes the advances undertaken until present, but also it identifies the unexplored pharmacological effects of this plant species. This step seems to be crucial for future investigations, especially for addressing the mechanisms of action of *G. alypum* extracts and/or its essential oils. More specifically, more than 150 articles that investigated *G. alypum*, published between 1991 and 2024, were reviewed. At first, the ethnomedicinal uses of the species were assessed, followed by exploration of its phytochemical composition, and its pharmacological activities. Finally, the main gaps in the knowledge of biological potentialities of this species and future steps to fill in these gaps were highlighted.

## 2. Literature Search Methodology

The present narrative review gathers all relevant information on botanical description, ethnobotanical usage, phytochemical components, and pharmacological and toxicological properties of *G. alypum*. A literature search across Web of Science, Scopus, PubMed, and other databases, covering studies published between 1991 and 2024 in English and French, was conducted. The literature search was updated till December 2024. The search employed specific keywords, namely “antioxidant activities”, “antidiabetic effects”, “antimicrobial effects”, “anticancer effects”, “anti-inflammatory effects”, “antiproliferative effects”, and “wounds healing effects” in conjunction with *G. alypum*. The investigation prioritized titles as the initial criterion for identifying and reviewing the relevant literature. In cases where ambiguity arose from the study’s title and abstract, a thorough examination of the full text was undertaken.

## 3. Taxonomy, Botanical Description, and Distribution

*Globularia alypum* belongs to the order Lamiales, family Plantaginaceae (Syn. Globulariaceae), and Tribu Globularieae Rchb. (1837). The family is formed of shrubs or sub-shrubs with zygomorphic flowers [10]. In Morocco, there is one genus and three species, namely *G. naini* Batt. and *G. liouvillei* Jahand. & Maire, both endemic [10,11], whereas the third one, *G. alypum* L., is common in shrublands of Mediterranean climate regions [10,12,13,76], and is native to Morocco, Algeria, Tunisia, Libya, Turkey, Albania, Baleares, Corse, East Aegean, Greece, Italy, Kriti, Sardinia, Sicilia, France, and Spain (Figure 1). *G. alypum* is a wild perennial evergreen shrub [10,13]. It has erected ligneous stems, its leaves are spatulate, lanceolate, or slightly tridentate at the apex; the flowers are purplish blue with a very short upper lip and perceptible and imbricate bracts [10,14]. It is a seeder and resprouting species [14]. In Morocco, *G. alypum* is found in *T. articulata* and *P. halepensis* Mill. forest stands, as well as in scrublands and matorrals, in Infra- and Meso-Mediterranean vegetation belts from the plains up to altitudes around 1800 m, under the hot and fresh variants of the semi-arid and subhumid bioclimates, and it often grows on calcareous and superficial soils [10].

## 4. Ethnomedicinal Uses

Table 1 lists the parts, mode of preparation, and therapeutic uses of *G. alypum*, commonly used in traditional Moroccan, Algerian, and Tunisian medicine [8,77,78]. Medicinal utilization is related to its used parts. Interestingly, the most widespread usage is linked to its antidiabetic properties [28,79]. Ouhaddou et al. reported that the infusion, decoction, and the powder of the whole plant of *G. alypum* in the Agadir Ida Ou Tanane province of Morocco has been commonly used against digestive disorders, skin infections, and respiratory and urinary tract diseases [80]. In the Northeast of Morocco, the decoction of *G. alypum* leaves has been recommended by the local population against diabetes [81], and to manage kidney diseases [82]. Another survey, proposed by Fakchich et al., highlighted the use of *G. alypum* decoction and infusion against diabetes, allergy, and digestives diseases [83]; on the hand, in the city of Agadir, El-Ghazouani et al. discussed the use of *G. alypum* for burns healing [84]. In Algeria *G. alypum* has been used for diabetes [77,85], but also as anti-jaundice [86], antihypertensive, astringent, digestive, sudorific, cholagogue, depurative, diuretic, laxative, and diaphoretic [36,87,88,89]. In the Taza province the decoction of *G. alypum* leaves has been reported against stomach pain, rheumatism, carminative, purgative, anxiolytic, and as menstrual-pain analgesic [90]. The infusion of *G. alypum* has been used instead by the Ksar Lakbir population for bilious stimulation [91] and against skin cancer [92]. Traditionally, *G. alypum* has been used primarily for the management of diabetes, digestive disorders, and skin ailments, with additional applications reported for urinary, circulatory, and inflammatory conditions. These dominant ethnomedicinal indications correlate closely with its main classes of bioactive metabolites: iridoids and phenylethanoid glycosides, which have demonstrated antidiabetic, anti-inflammatory, and antioxidant activities; flavonoids, which are associated with antioxidant and skin-protective effects; and terpenoids, which may contribute to digestive and antimicrobial properties. This integrative perspective underscores a clear link between traditional knowledge and the pharmacological potential of the plant, supporting the rationale for its continued use and further experimental investigation.

## 5. Phytoconstituents of *G. alypum*

*G. alypum* phytochemistry was extensively studied in the literature (Table 2 and Table 3). The main focus was on organic extracts chemical profiling of various plant parts (leaves, flowers, stem, and underground parts). An overview of the peer-reviewed literature is summarized in Table 2. Friščić et al. performed a comparative phytochemical study of four *Globularia* species collected in Croatia. These authors reported that the level of all secondary metabolites is significantly (*p* < 0.05) higher in *G. punctata*, *G. cordifolia*, and *G. meridionalis* as compared to those of *G. alypum*. According to the same authors, *G. punctata* contains the greatest concentration of total flavonoids (48.49–63.03 mg QE/g DE) as well as iridoids (343.33–440.04 mg AE/g dry extract (DE)). With respect to the other studied species, the aerial parts from *G. alypum* contained the highest amount of TPC (112.34 mg GAE/g DE). No significant variations (*p* > 0.05) in TPC and TFC were observed between *G. cordifolia* and *G. meridionalis* extracts. As demonstrated by Friščić et al., *G. alypum* is a rich source of flavonoids, iridoids, lignans, and phenylethanoids among others. Major metabolites were represented by globularin and verbascoside [124]. Some works, carried out by liquid chromatography in combination with mass spectrometry (LC-MS) highlighted important quantitative differences in major compounds in the leaf and aerial part extracts; considering the shrubby nature of this plant, such variations could be assigned partially to a relatively high share of stems of *G. alypum* aerial parts subjected to extraction. Also, the stems of *G. alypum* were found to be less rich in terms of secondary metabolites than their leaves and flowers [50,125,126,127]. In a study by Kırmızıbekmez et al., three new phenylethyl glycosides were isolated from *G. alypum* leaves, along with two known phenylethyl glycosides, calceolarioside A and verbascoside, and eight iridoid glucosides, all identified by 1D- and 2D-NMR and HR-MALDI-MS [47].

Friščić et al. employed LC-MS/MS for the analysis of the methanolic extracts of the aerial parts obtained from four *Globularia* species leading to the identification of up to 85 compounds, mainly represented by globularin, globularifolin, asperuloside, and verbascoside. While studying hypoglycemic and hypolipidemic activities of *G. alypum* leaves, collected in Achba-Tlemcen, Algeria, during the species fructification period (July), Merghache et al. (2023) isolated globularin, an iridoid glucoside, from the plant leaves (3.4%) [128]. Likewise, Sertić et al. used a similar analytical platform for the analysis of the methanolic extracts from four *Globularia* species. Two iridoid glucosides, viz. aucubin and catalpol, were positively identified. Flowers had the greatest amount of investigated iridoids and catalpol content being 1.6% in *G. punctata* flowers. Compared *to G. alypum*, the other related species presented a major amount of the iridoid content [129]. Finally, Mamoucha et al. employed UHPLC-HRMS analysis for the elucidation of the chemical pattern and seasonal variations of *G. alypum* collected in Greece. A total of 24 compounds belonging to iridoids, flavonoids, and phenylethanoid glycosides were positively identified. Regarding iridoids, (E)-globularicisin was the most abundant one (up to 70%). According to the same authors, total iridoid was raised by 48.1% in summer. In a similar trend, flavonoid anabolism was more active for the same period, with less noticeable seasonal variation accounting for about 25.6%. On the other hand, a sharp fall (33.5%) of phenylethanoids was observed for the summer season [48]. Based on the compiled data, iridoids such as globularin, globularifolin, and aucubin/catalpol appear to be the most consistent candidate marker compounds distinguishing *G. alypum* from other *Globularia* species. In addition, the relative abundance of phenylpropanoid derivatives and certain flavonoids suggests a chemotype tendency characteristic of *G. alypum* within the genus.

**Table 2:** Phytoconstituents of *G. alypum* extracts.

Plant Part	Region	Chemical Composition of Organic Extracts/Essential Oil (Major Components)	References
**Leaves, flowers, woody stems, and underground parts of four *G. alypum* L. species**	Croatia and Bosnia and Herzegovina	Aucubin (0.58–0.92 mg/g DM) in ME Catalpol (1.79–12.85 mg/g DM) in ME	[129]
**Leaves**	Algeria (Bajaia)	Hydroxyluteolin 7-Olaminaribioside, Gallocatechin/ Epigallocatechin, Eriodictiol 7-O-sophoroside, Quercetin glucoside, Luteolin sophoroside, Cynaroside, Amurensin, Nepitrin, Phellamurin.	[130]
**Aerial parts**	Morocco (Taza)	Syringin, four phenylethanoids, four flavonoids and six iridoids	[45]
**Aerial parts**	Morocco (Taza)	Iridoids globularin, globularicisin, globularidin, globularinin, globularimin, chlorinated iridoid glucoside (globularioside)	[37]
**Leaves**	Tunisia (Gafsa)	Decaffeoylverbascoside, Caffeoyl hexoside 1, 6-O-caffeoyl-b-D-glucopyranosyl-(1-6)-glucitol (hebitol II), Caffeoyl hexoside 2, Syringin, Caffeoyl hexoside, Coumaroyl hexosyl glucitol, 6-O-caffeoyl-3,4-dihydrocatalpol, (dihydroverminoside), 6-O-feruloyl-b-D-glucopyranosyl-(1-6)-glucitol (globularitol), 6-O-Caffeoylcatalpol, 6-Hydroxyluteolin 7-O-laminaribioside, Specioside, 6-Hydroxyluteolin 7-O-glucoside, Luteolin di-hexoside, Globularinin, Globularimin, Calceolarioside A/calceolarioside B, Rossicaside A, Luteolin 7-O-glucoside (cynaroside), Verbascoside or acteoside isomer 1, Calceolarioside A/calceolarioside B, Caffeoyl hexoside derivative, 6-Methoxyluteolin-7-O-glucoside (nepitrin), Globularicisin, Methylcaffeoyl derivative, Verbascoside or acteoside isomer 2, Globularidin, Hydroxyphenylethyl-caffeoyl-hexoside, Diydroxyphenylethyl-coumaroyl-hexoside, Globularin, Dihydroxyphenylethyl-methylcaffeoyl-hexoside, 6-Methoxyluteolin (nepetin), Martynoside, Globularioside, Hydroxyphenylethyl-methylcaffeoyl-hexoside, 6-O-caffeoylverbascoside, Galypumoside A, Galypumoside B, Galypumoside C	[131]
**Aerial parts**	Croatia (Konavle cliffs, Grobnik field, Baške Oštarije, Velebit, Alan, Velebit)	**Leaves extracts** TPC = 130.46 ± 5.99–131.39 ± 2.89 mg GAE/g dry extract (DE, ME) using ultrasound-assisted extraction (UAE) TFC = 30.43 ± 0.29- 32.26 ± 1.37 mg QE/g DE (ME) using UAE Iridoids 12.07 ± 0.18–27.49 ± 3.08 mg aucubin equivalents (AE)/g DE (ME) using UAE Condensed tannins: 2.66 ± 0.09–3.00 ± 0.06 mg catechin equivalents (CE)/g DE using UAE **Aerial parts (flowers and stems) extracts** TPC = 112.34 ± 2.17 mg GAE/g DE ME using Soxhlet extraction. TFC = 26.85 ± 0.46 mg QE/g DE ME using Soxhlet extraction. **LC-MS profile for Methanolic leaf extracts (UAE) from *G. alypum*** Mannitol, sucrose, catalpol, aucubin, 1′-O-Hydroxytyrosol glucoside, Caffeoylglucoside isomer, Hebitol II (6′ -O-Caffeoyl-β-D-glucopyranosyl-(1→6)-mannitol), Globularitol (6′ -O-Feruloyl-β-D-glucopyranosyl-(1→6)-mannitol), Verminoside (6-O-Caffeoylcatalpol), Geniposide, Vicenin-2 (Apigenin-6,8-di-C-glucoside), Specioside (6-O-(p-Coumaroyl)-catalpol), 6-Hydroxyluteolin 7-O-sophoroside, 6-Hydroxyluteolin 7-O-glucoside, Alpinoside, Globularinin, Globularimin, Liriodendrin ((+)-Syringaresinol di-O-β-glucopyranoside), Isoquercitrin (Quercetin 3-O-glucoside), Calceolarioside A, Calceolarioside B, Nepetin 7-O-glucoside, Rossicaside A, Verbascoside, Globularidin, Isoverbascoside, Forsythoside A, Globularin (10-O-trans-Cinnamoylcatalpol), Leucosceptoside A, 6′ -O-Feruloyl-10 -O-hydroxytyrosol glucoside, Globularioside, Alpinoside-alpinoside dimer, Globusintenoside isomer, Desrhamnosyl 6′ -O-caffeoylverbascoside, 6′ -O-Caffeoylverbascoside, Globuloside A (Alpinoside-globularin dimer), Galypumoside B (6′ -O-Feruloylverbascoside), Desrhamnosyl galypumoside B, Oxo-dihydroxy-octadecenoic acid, Galypumoside C, Trihydroxy-octadecenoic acid **LC-MS profile for Methanolic aerial parts extracts (Soxhlet) from *G. alypum*** Mannitol, sucrose, catalpol, Caffeoylglucoside isomer, Gardoside, Verminoside (6-O-Caffeoylcatalpol), Geniposide, Specioside, 6-Hydroxyluteolin 7-O-sophoroside, 6-Hydroxyluteolin 7-O-glucoside, Alpinoside, Globularinin, Globularimin, Liriodendrin ((+)-Syringaresinol di-O-β-glucopyranoside), Calceolarioside A (Desrhamnosyl verbascoside), Calceolarioside B, Rossicaside A, Verbascoside, Isoverbascoside, Forsythoside A, Globularin (10-O-trans-Cinnamoylcatalpol), Leucosceptoside A, 6′ -O-Feruloyl-10 -O-hydroxytyrosol glucoside, Globularioside, Globusintenoside isomer, 6′ -O-Caffeoylverbascoside, Apigenin, Globuloside A, Galypumoside B	[125]
**Fresh leaves**	Tunisia (Beja region)	Yield (%) 14.20 ± 0.28, total sugars (%) 71.56 ± 0.64, Sulfate (%) 13.29 ± 0.84 Proteins (%) 0.74 ± 0.02, Lipids 1.45 ± 0.08, Moisture (%) 7.53 ± 0.35, Ash (%) 6.8 ± 0.62	[132]
**Fresh leaves**	Tunisia (Beja region)	**Methanolic extracts**: Glycerol, tris (TMS) ether (2.65%), Cinnamic acid, TMS ester (1.53%), Cinnamic acid (1.32%), Cinnamic acid, (E) (0.32%), Cinnamic acid, TMS ester (23.21%), 4-Hydroxyphenylethanol, di-TMS (4.70%), Ethanol, (2-(3,4-dihydroxyphenyl)-, tris(TMS) (6.61%), D-Fructose, 1,3,4,5,6-pentakis-O- (TMS) (12.24%), 3,4-Heptadien-2-one, 3,5-dicyclopentyl-6-methyl- (12.46%), Bicyclo [4.4.0]dec-2-ene-4-ol, 2-methyl-9-(prop-1-en-3-ol-2-yl) (30.96%), Talose, 2,3,4,5,6-pentakis-O-(TMS)- (0.62%), Palmitic acid, TMS ester (6.07%), Silane, [(3,7,11,15-tetramethyl-2-hexadecenyl)oxy] TMS (0.47%), Linoleic acid, TMS ester (0.40%), Oleic acid, TMS ester (1.48%), Stearic acid, TMS ester (0.75%), 1-(3′, 5′ -dichlorophenyl)-5-oxo-4,4-diphenyl-2-imidazolin-2-yl] guanidine (0.3%)	[40]
**Dried aerial parts**	Croatia	α-himachalene (35.34%), β-himachalene (13.62%), γ-himachalene (12.6%), cedrol (10.32%), isocedranol (5.52%) and α-pinene (5.5%)	[128]
**Aerial part (flowers, leaves and stems)**	Algeria (Batna)	Diethyl ether (TPC = 331.88 ± 17.80 µg GAE/mg DE), TFC = 223.46 ± 2.37 µg CE/mg DE) Ethyl acetate (TPC = 103.62 ± 1.09 µg GAE/mg DE), TFC = 139.58 ± 4.08 µg CE/mg DE) n-butanol (TPC = 127.70 ± 0.82 µg GAE/mg DE), TFC = 173.5 ± 4.71 µg CE/mg DE) Gallic acid, p-coumaric acid, rutin, naringenin and quercitin (**diethyl ether fraction**) Gallic acid, quercetin, rutin, and ferulic acid **(ethyl acetate fraction)**	[42]
**Aerial (flowers, leaves, and woody stems) and underground parts**	Croatia, Bosnia and Herzegovina	**Methanolic extracts (ultrasonic extraction)** Aucubin (mg/g DE): Leaves 0.58 ± 0.01, Flowers 0.92 ± 0.00, Woody stems 0.58 ± 0.01 Catalpol (mg/g DE): Leaves 1.79 ± 0.03, Flowers 12.58 ± 0.03, Woody stems 1.47 ± 0.02	[129]
**Fresh aerial parts**	Morocco (Taza)	**Aqueous MeOH extract**: **Phenolics**: 6-hydroxyluteolin 7-O-laminaribioside, eriodictyol 7-O-sophoroside, and 6′- O-coumaroyl-1′-O-[2-(3,4-dihydroxyphenyl) ethyl]-β-D-glucopyranoside, **Phenylethanoid glycosides**: acteoside, isoacteoside, and forsythiaside **Flavonoid glycosides**: 6-hydroxyluteolin 7-O-β-D-glucopyranoside and luteolin 7-O-sophoroside	[13]
**Fresh leaves**	Morocco (Taza)	**Ethyl acetate**: TPC = 56.5 ± 0.61 µg GAE/mg of extract, TFC = 30.2 ± 0.55 µg CE/mg of extract **Chloroform**: TPC = 18.9 ± 0.48 µg GAE/mg of extract, TFC = 18.0 ± 0.36 µg CE/mg of extract **n-hexane fraction (CG-MS)**: A total of 73 compounds: the major compounds were n-hexadecanoic acid (13.5%), oleic acid (12.98%), and linoleic acid (11.58%) **Ethyl acetate extract (HPLC-DAD-ESI/MS):** Gallic acid (4.3 ± 0.12 mg/100 g), Gallic acid ethyl ester (4.2 ± 0.20 mg/100 g), Quercetin glucoside (4.7 ± 0.20 mg/100 g), Quercetin rhamnoside (14.5 ± 1.20 mg/100 g), Kaempferol derivative (10.8 ± 0.50 mg/100 g), Quercetin acetyl hexoside (0.3 ± 0.01 mg/100 g), Quercetin glucoside (7.8 ± 0.30 mg/100 g)	[3]
**Aerial parts**	Tunisia (Seliana)	**Ethanolic extracts** TPC = 23.95 ± 0.24 (mg GAE/g DE), TFC = 11.93 ± 0.05 (mg CE/g DE) and TCT = 21.43 ± 0.38 (mg CE/g DE) RP-HPLC/UV of ethanolic extracts: **Phenolic acids**: p-coumaric acid (15.02 ± 1.55 mg/g DE), Sinapic acid (3.62 ± 0.03 mg/g DE), Trans-hydroxycinnamic acid (34.21 ± 3.24 mg/g DE) **Flavonoids**: Quercetin (0.11 ± 0.00 mg/g DE), Catechin hydrate (16.01 ± 1.87 mg/g DE)	[133]
**Dried whole plant**	Switzerland	Methanolic/aqueous extracts: globularicisin, globularidin, globularimin, globularinin, lignan diglucoside liriodendrin, syringin, globularin, catalpol	[134]
**Fresh leaves**	Algeria (Souk Ahras)	Petroleum ether extract (GC-MS): Ethylbenzene, Xylene, D-Fenchone, Camphor, alpha-Terpineol, Neohexane, alpha-Fenchyl acetate, n-Tetradecane, Sabinyl acetate, 1-Ethyl-1,5-cyclooctadiene, Eugenol, Isoeugenol, Diethylmethyl-borane, Eicosane, 17-Pentatriacontene, Nonadecane, Pentadecane, n-Octadecyl chloride, Bicyclopentyl-2′-en-2-yl-dimethylamine, 3-Cyclopentyl-pentane, Lignocerol, Viridiflorol, Cetane, alpha-Cadinol, Aromadendrene, 2,2-Dimethyl-6,10-dithiaspiro [4.5] decan-1-ol, Hexatriacontane, Neoclovene, Dehydroionone Amphetamine oxime acetate, Phthalic acid	[135]
**Dried leaves**	Tunisia (Boussalem)	Aqueous extract: Catalpol, Shanzhizide, Hebitol, Globuralitol, Varminoside, Gallocatechin, Quercetin-glucoside, Verbascoside, Isoverbascoside, Decumbeside, Phellamurin, Serratoside, Globularioside	[46]
**Leaves**	Tunisia (Zaghouan)	Ethanolic extracts: TPC 25.33 ± 1.52 mg GAE/g DW, TFC 4.71 ± 0.50 mg CE/g DW, tannins 4.30 ± 0.35 mg CE/g DW HPLC-PDA-ESI-MS/MS: p-coumaroyl sugar ester, Apigenin acetoxydeoxyhexose, Tetragalloyl hexoside, HHDP-hexose, Sinapic acid derivative, Apigenin glucuronide, Amentoflavone, I3, II8-Biapigenin, Myricetin, Kaempfreol glucoside, Naringenin diglucoside	[136]
**Leaves**	Greece (Athens)	**UHPLC- (-) ESI/HRMS**: Gardoside/Geniposidic Acid, Cornoside, Mussaenosidic acid, (epi)loganic acid, Secoxyloganin methylester, Acetylbarlerin, Globularitol, Hydroxyluteolin diglucoside, Eriodictiol diglycoside, Luteolin diglycoside, Quercetin glucoside, Alpinoside, Verbascoside, Agnuside, Globularinin, Calceolarioside B, Isoverbascoside, Nepitrin, Gallocatechin/Epigallo- catechin, (Z)-Globularicisin, Globularidin, (E)-Globularicisin, Globularioside/Baldac-cioside, Verminoside	[48]

**Table 3:** Representative bioactive compounds of *Globularia alypum* showing their 2D and 3D structures.

Compound Name	2D Structure	3D Structure
Globularin	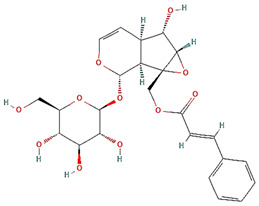	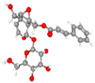
Globularifolin	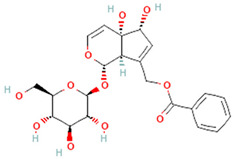	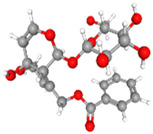
Catalpol	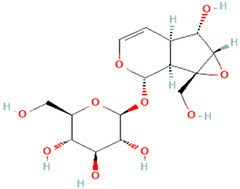	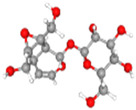
Aucubin	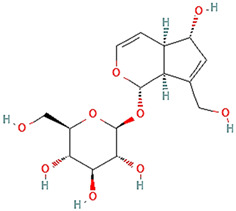	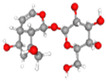
Verbascoside	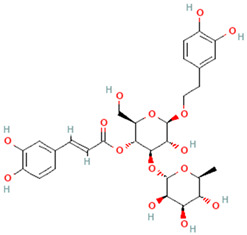	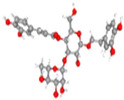

## 6. Pharmacological Activities

### 6.1. Antioxidant Activity

Oxidative processes, such as energy production, fat metabolism, alcohol and drug detoxification, and digestion, naturally generate free radicals in the human body. While endogenous antioxidant systems work to neutralize these species, excessive accumulation may disrupt cellular processes, damage membranes and DNA, and impair energy metabolism [137]. Oxidative stress has been associated with the progression of various metabolic, chronic disorders, and cancers [138,139,140]. Antioxidants are molecules capable of stabilizing free radicals, thereby limiting their potential harmful effects [141,142].

As summarized in Table 4, extracts from different parts, namely leaves, fruit, and seeds, of *G. alypum* have been explored through DPPH, FRAP, β-carotene, and ABTS. In a vitro study, Asraoui et al. studied the antioxidant capacity of the organics extracts of Moroccan *G. alypum* using DPPH, ABTS, and FRAP assays. The crude extracts from *G. alypum* leaves collected from Taza region revealed attractive antioxidant effect (IC_50_ = 12.3 ± 3.83 µg/mL for DPPH test, IC_50_ = 37.0 ± 2.45 µg/mL for ABTS test, and 531.1 ± 1.08 mg AAE/g DW for FRAP test) for the EtOAc extract [3]. Such results agree with the study by Khantouche et al. on the ethanolic extract of Tunisian *G. alypum* leaves expressing an IC_50_ value of 29.8 ± 0.20 µg/mL and 42.53 ± 0.24 µg/mL, respectively, for DPPH and β-carotene assays; however, the ABTS assay showed a value of 89.5 ± 0.32 mg EVC/g DW, compared to Vitamin C, Vitamin E, and BHT [143]. Another work revealed that the diethyl ether extract from the aerial part of *G. alypum* yielded an antioxidant activity, corresponding to an IC_50_ value of 20.54 ± 0.48 µg/mL and an EC_50_ value of 283.7 ± 82.59 µg/mL for DPPH and FRAP tests, respectively, lower with respect to the standard ascorbic acid (IC_50_ = 3.74 ± 0.11 µg/mL, EC_50_ = 79.3 ± 2.08 µg/mL, respectively) [42]. A remarkable anti-DPPH effect (IC_50_ = 4.95 ± 0.94 µg/mL) compared to ascorbic acid (IC_50_ = 0.89 ± 0.06 mg/mL) was found in a study conducted by Touaibia et al. on leaves ethanolic extracts [40]. In another study, focused on the roots, leaves, and steams ethyl acetate and methanolic extracts of *G. alypum*, harvested from Taounat region of Morocco, IC_50_ values as high as 33.67 µg/mL, 48.28 µg/mL, and 18.85 µg/mL, respectively, compared with the positive control BHT (3.32 µg/mL) were obtained. Roots extract of *G. alypum* yielded a higher IC_50_ value followed by steams, and leaves (10.01 µg/mL vs. 22.11 µg/mL vs. 25.65 µg/mL) [144]. Feriani et al. reported, for the methanolic extract of *G. alypum* leaves, a moderate anti-DPPH activity with an IC_50_ value of 203 ± 1.83 µg/mL compared to IC_50_ = 168.85 ± 2.05 µg/mL and an EC_50_ of 15.25 ± 0.49 mg/mL for ABTS and reducing power, respectively [131]. In addition to leaves, flowers of *G. alypum* were investigated by Chograni et al. [14], reporting RSA and FRAP values ranging from 230 to 295 µmol TEAC/g and from 24.27 to 21.81 mmol Fe^2+^/L, respectively. Notably, different extracts, namely aqueous, methanol, petroleum ether, and ethyl acetate extracts from *G. alypum* leaves, were exploited by Harzallah et al. [19], highlighting the major antioxidant results for the aqueous extract (8.9 mM TE).

### 6.2. Antimicrobial Activity

Excessive and incorrect antibiotic usage has led to antimicrobial resistance, a significant global concern and threat to humanity. Traditional pharmacological therapies are losing effectiveness, prompting the development of new antimicrobial medicines based on natural metabolites for their chemical variety and efficacy. They suppress pathogen growth, including bacteria, fungi, and viruses, and enhance antibiotic efficacy, potentially overcoming antimicrobial resistance [149]. For this reason, different extracts of *G. alypum* have been subjected to several antimicrobial investigations.

Table 4 summarizes all studies that studied the antibacterial and antifungal activity of *G. alypum*, including the used part, type of extract, antimicrobial methods, tested microorganisms, and results. Various works showed the antibacterial potential of organic extracts and essential oil from different parts of *G. alypum*; Krimat et al. evaluated in vitro the antibacterial activity of hydroethanolic extract of *G. alypum* collected from Setif, Algeria against four bacterial strains and one yeast. Notably, *B. subtilis*, *S. aureus*, and *C. albicans* turned out to be the most sensitive to the extract with a diameter of inhibition zone ranging from 9.0 to 11.0 mm. However, no inhibitory effect was observed for *E. coli* and *P. aeruginosa* [1]. A larger study was carried out by Boussoualim et al., who studied the antibacterial effect of Algerian *G. alypum* different extracts against 11 bacterial strains (*P. aeruginosa*, *E. coli*, *S. typhimurium*, *A. baumanii*, *C. freundii*, *P. mirabilis*, *K. pneumoniae*, *S. aureus*, *B. cereus*, *E. faecalis*, and *L. monocytogenes*). Methanol (CrE), chloroform (ChE), and ethyl acetate (AcE) extracts revealed the highly inhibitory potential; particularly, ChE and AcE extracts were found the most active. Aqueous (AqE) extracts presented a poor effect of inhibition. AcE and CrE were the most active towards *S. aureus*, with an inhibition zone diameter of 16 and 15 mm, respectively; on the other hand, ChE against *S. aureus* had an inhibition zone diameter of 13 mm, whereas 12 mm was obtained towards *A. baumanii.* The lowest effect on the totality of bacteria was attained for the aqueous extract, except towards *K. pneumoniae* [150]. Ghlissi et al. examined the antibacterial activity of leaves methanolic extracts by means of agar-well diffusion assay against six microorganisms, three Gram-positive and three Gram-negative bacteria species, at a concentration of 50 mg/mL, using ampicillin as positive control. The potent antibacterial activity could be explained by the dominance of cinnamic acid compounds (26.38%), and in particular, the occurrence of cinnamic acid-TMS ester (23.21%) [40]. The aqueous extracts of *G. alypum*, obtained by four extraction methods, against four bacterial strains, isolated from patients with urinary infections, compared to antibiotics (*Kanamicin*, *Colistin*, *Amoxillin*, *Gentamicin* and *Ampicillin*), served as standards and was carried out by Bouabdelli et al. The bacterial strains tested were *E. coli*, *P. mirabilis*, *P. aeruginosa*, and *S. aureus* using the disc diffusion method. The results demonstrated that all the extracts showed a positive effect, and all the tested bacteria were sensitive to *G. alypum* extracts with an inhibition zone diameter ranging from 7 mm to 15 mm with a concentration of 50 mg/mL. Such results highlight the importance of the method extraction on the antibacterial potential of plant extracts [151]. Another study proved the antibacterial properties of the ethyl acetate and methanol extracts from different parts of plant (leaves, stems, and root) against selected bacteria strains. Notably, only the ethyl acetate extracts showed inhibitory effects against *M. luteus*. In addition, the ethyl acetate extract of roots turned out to be the most active with a MIC value of 0.5 mg/mL against both bacterial strains; on the other hand, the MBC values of the extracts were equal to the MIC values, showing a positive bactericidal effect. *B. subtilis* was more susceptible to the methanolic extracts of roots and stems, differently from *S. aureus*, being more sensitive to leaves methanolic extract [144]. An interesting work carried out on the essential oil obtained from the aerial parts of *G. alypum* collected by two localities against seven Gram negative bacteria and five Gram positive bacteria using macro-dilution assay was reported by Ramdani et al. The results showed a variable antimicrobial effect of the tested oil against all clinical bacteria pathogens. The essential oil of Boutaleb and Khenchela populations presented important potential against Gram-negative bacteria with a moderate inhibition against *A. baumanii*. A low activity was obtained against *K. pneumoniae* and *P. mirabilis*, whereas *S. aureus*, *B. cereus*, *K. pneumoniae*, and *P. mirabilis* were found to be resistant to this essential oil [55]. The antimicrobial and antifungal effects of the hydro-alcoholic and aqueous leaf extracts of *G. alypum* collected from the region of Laghouat, Algeria, was investigated by Kraza et al. Up to five bacterial strains (*S. aureus*, *E. faecalis*, *P. aeruginosa*, *E. coli*, and *S. typhi*) and three pathogenic fungal strains (*Aspergillus flavus*, *Penicillium* spp., and *Fusarium oxysporum*) were tested and the authors reported the inhibition of the growth against strains tested of ethyl acetate, butanol, and aqueous extracts at different concentrations (50, 60, 80, and 100 mg/mL). The antibacterial effect was proportional to the concentration of the extract, and the ethyl acetate extract exhibited meaningly the highest antibacterial action against all pathogenic bacteria, with a maximum inhibition zone (15.5 ± 0.2 against *P. aeruginosa*) [41].

The antibacterial potential of *G. alypum* of methanolic extracts of aerial parts obtained by Soxhlet extraction was reported by Frišcic et al. Considering the former method, *G. alypum* had the highest inhibitory activity against *S. aureus* (25.0 mm), whereas lower against *B. cereus*, *B. subtilis*, and *P. aeruginosa* (8.3 mm, 7.7 mm, and 9.7 mm); on the other hand, considering the latter, the assessed MIC value against S. aureus ATCC 6538 was reported with a MIC of 1.42 mg/mL, whereas a comparable antibacterial activity was attained against *methicillin*-resistant *S. aureus* (MIC = 2–4 mg/mL) [125]. Recently, research conducted by Asraoui et al. examined the antifungal and antibiofilm properties of leaf extracts from of *G. alypum* against *Candida*, both in vitro and in vivo. Such a study assessed the ability of these extracts to inhibit the growth of *Candida albicans* and *Candida glabrata* in their planktonic and sessile forms. Additionally, an in vivo infection model involving *Galleria mellonella* larvae infected with *Candida* and treated with the extracts was conducted. All tested extracts exhibited strong fungicidal activity with minimum fungicidal concentrations (MFC) ranging from 128 to 512 μg/mL. It is noteworthy that the methanolic extracts demonstrated the lowest MFC value (128 μg/mL), capable of effectively inhibiting 90% of biofilm formation. In vivo experiments on *Galleria mellonella* larvae indicated that the extracts might enhance the survival rate of larvae infected with *Candida*, holding promise as novel antifungal and biofilm-inhibiting agents for applications in pharmaceuticals and agro-food industries [152]. The differences in the inhibition zone diameter (expressed in mm), along with MICs, and MBCs values of the extracts versus a varied range of bacterial and fungal strains, listed in Table 5, could be explained by the difference in the chemical composition of the different extracts. In particular, the used solvent, the plant part, and the origin of species significantly impacted the variety and the level of bioactive compounds and consequently might affect the antimicrobial capacity.

### 6.3. Anti-Hyperglycemic Activity

Oral anti-hyperglycemic agents are often associated with adverse effects, leading sometimes in drug discontinuation [153]. In such a context, phytotherapy provides a valuable opportunity for the discovery of novel natural molecules capable of avoiding the harmful effects of synthetic therapeutic agents.

*G. alypum* is described as a well-known antidiabetic plant in traditional medicine in Morocco. The leaves of this plant are usually prepared by infusion or decoction (according to the region) [8,21,24,27,31,83,154,155]. The antidiabetic effect of *G. alypum* extracts was reported by in vitro investigations of several researchers showing how the main compounds do possess antidiabetic effect by increasing insulin secretion in addition to controlling the synthesis of hepatic glycogen, and the regulation of glycemia.

Ouffai et al. evaluated the inhibitory effect of several *G. alypum* extracts against α-amylase and α-glucosidase. The diethyl ether fraction yielded the most satisfactory inhibitory properties against both α-amylase (IC_50_ = 0.57 ± 0.05 mg/mL) and α-glucosidase (IC_50_ = 0.52 ± 0.02 mg/mL) with respect to acarbose served as a positive control. Such effects, especially on insulin secretion, were correlated to the phenolic content of the plant extracts [42].

Zennaki et al. evaluated the antidiabetic activity of the aerial parts extracts of *G. alypum*. In STZ-induced diabetic rats, the administration of *G. alypum* extract significantly reduced blood glucose and glycosylated hemoglobin levels. The treatment with the *G. alypum* methanolic extract revealed a notable reduction in GSH levels in liver, brain, and kidney [34].

In 2014, Djellouli et al. evaluated the effects of *G. alypum* methanolic extract on glycaemia, and reverse cholesterol transport, in addition to lipoprotein peroxidation in STZ-induced diabetic rats. As a result, they found that after 28 days of treatment with the methanolic extract of *G. alypum*, there was a notable hypoglycemic effect; however, the blood glucose level reached a mean value of 5.32 ± 0.15 mmol/L, in comparison to 27.83 ± 0.41 mmol/L obtained in the diabetic group. The increased serum insulin levels in *G. alypum* treated diabetic rats was inferred by the authors to the insulinotropic molecules occurring in *G. alypum* extract that persuade the whole functional β-cells of the Langerhans islet to produce insulin [4]. Skim et al. studied the hypoglycemic effect of the aqueous extract of *G. alypum* leaves alloxan-diabetic rats. The infusion of *G. alypum* leaves demonstrated a hypoglycemic effect after both oral and intraperitoneal administration. Such an effect was like common hypoglycemic compounds, e.g., metformin and glibenclamide, at a concentration of 11.3 and 0.13 mg/kg, respectively [54]. Merghache et al. studied the hypoglycemic effect of globularin isolated from *G. alypum*. Notably, a single intraperitoneal administration of globularin at a dose 100 mg/kg body weight produced the maximum decrease in blood glucose levels [51]. In a recent study, Tiss et al. assessed the in vitro antidiabetic effect of *G. alypum* extracts (methanol and water), via the inhibition of α-amylase activity. Notably, the methanolic extract exhibited the highest anti-α-amylase activity with half inhibitory concentration IC_50_ = 2.97 mg/mL [156]. On the other hand, the study of Jouad et al. demonstrated that oral administration of the aqueous extract of *G. alypum* leaves did not change blood glucose levels in normal rats, differently from streptozotocin (STZ) diabetic rats, where a significant decrease in blood glucose levels was noticed [157]. Another recent study of Frišcic et al. reported the effect of *G. alypum* methanolic extracts against α-glucosidase activity, and the results showed the effectiveness of *G. alypum* at the concentrations tested (0.5 and 1.0 mg/mL) with a percentage of inhibition ranging from 30.0 to 45.7% [125].

A thorough work was carried out by Tiss and Hamden on the effect of *G. alypum* methanolic and aqueous leaves extracts on hyperglycemia, pancreas, liver, kidney, testes, and bone tissue.

The pancreas tissue was destroyed after administration of alloxan, and despite this, destruction was minimized in GAWE and GAME diabetic-treated rats [158].

### 6.4. Anti-Inflammatory Activity

Inflammation is an important process in the immune system that plays a crucial role in the body’s defense mechanism against harmful stimuli [159]. However, when inflammation becomes excessive, it can lead to health problems and the development of various diseases [160]. In the last case, the inhibition of inflammation represents a physiological mechanism that is naturally implicated or can be targeted via treatments. This process is of high importance to the treatment of inflammatory diseases or illnesses linked to excessive inflammation such as cancer and autoimmune diseases [161,162]. Vegetables and fruits, rich in antioxidants and polyphenols, showed a great ability to protect against inflammation [163,164]. In addition, a considerable number of medicinal plants, such as Allium sativum and Pinus pinaster, are known for their anti-inflammatory activity as a source of anti-inflammatory compounds [165,166,167]. *G. alypum* is one of the medicinal plant species of the Mediterranean region that possesses an anti-inflammatory activity (Table 6). Ethnobotanical studies reported that *G. alypum* is used in the treatment of many diseases related to inflammation such as rheumatism, arthritis, eczema, and colic [17,35,168]. In addition, this species is used in the treatment of burns and wounds by the Moroccan population [84,136].

Studies showed that *G. alypum* has an important anti-inflammatory effect through the reduction in lipid peroxidation, pro-inflammatory cytokines (IL-6 and TNF-α) levels, nuclear factor NF-kB expression and cyclooxygenase and lipoxygenase (key-enzymes linked to pain and inflammation) activity, with the amelioration of the reverse cholesterol transport [4,56,169,170]. In addition, *G. alypum* significantly decreases the C-reactive protein levels, used as a marker of inflammation [40]. As results, *G. alypum* extracts have been found able to prevent lipid disorder in STZ-induced diabetic and in hypercholesterolemia rats [4,169], as well as showing an anti-inflammatory effect on human colon [170]. Moreover, oral administration of *G. alypum* extract in rats induced a noticeable reduction in the ulcer index (UI) of gastric lesions produced by EtOH-HCl from 2.66 to 1.66 mm, resulting in recovery of the gastric mucosa with a percentage of protection of 30% and a healing rating of 37.59% [133].

In fact, the methanolic extract of *G. alypum* leaves showed a potent inhibitory activity against 5-lipoxygenase enzyme with an IC_50_ value of 79 mg/L [56], and against cyclooxygenase-1 enzyme with a percentage inhibition of 51.3% in the TMPD assay and 40.6% in the PGE2 assay at a concentration of 50 µg/mL [125]. In contrast, another study showed that the methanolic extract of *G. alypum* leaves presented a weak inflammatory activity, with a cyclooxygenase-1 inhibition by only 5.33% at a concentration of 33 µg/mL, while the flowers showed a potent inhibition of cyclooxygenase-1 enzyme by 61.05% with an IC_50_ value of 0.122 mg/mL at the same concentration (33 µg/mL) [35]. To our knowledge, there are no further studies comparing the anti-inflammatory activity of *G. alypum* leaves and flowers to confirm the results of Amessis-Ouchemoukh and collaborators.

The methanolic extract of *G. alypum* leaves inhibited inducible nitric oxide synthase (iNOS) expression and NO production by 45% at a concentration of 150 mg/L and by 66% at 600 mg/L in LPS-stimulated RAW 264.7 macrophages [56]. However, Ben Mansour et al. found that the ethanolic extract of *G. alypum* leaves has a moderate inhibitory effect on NO generation (29% at a concentration of 200 mg/mL) in comparison to methanolic extract [133]. The anti-inflammatory activity of *G. alypum* was tested in vivo against carrageenan-induced paw oedema. The results showed a significant reduction in oedema size and swelling in the groups treated with *G. alypum* extracts in comparison to the control groups in a dose- and time-depending manner [40,57,171]. The methanolic extracts of the aerial part of *G. alypum* decreased the oedema volume from 64 to 28 μL with a percentage inhibition of 56.2% at a concentration of 500 mg/kg in mice [171], while at a concentration of 200 mg/kg the paw size deceased significantly by 40% in rats [40]. In addition, the flavonic extract of *G. alypum* exhibited a percentage inhibition of paw oedema of 33.01% at a concentration of 0.3 g/mL in mice [57].

The anti-inflammatory effect of *G. alypum* could be linked to many compounds detected in their extracts and that are known for their anti-inflammatory activity such as cinnamic acids [172], catalpol and its derivatives like globularin [173], liriodendrin [174], and verbascoside [175]. However, as in most medicinal plants, the chemical composition of *G. alypum* might change according to different factors such as season, geographical origin, environment conditions, and part of plant, which explains the heterogeneity in obtained results in terms of efficiency according to plant part and chemical composition.

**Table 6:** Anti-inflammatory results of *G. alypum* extracts.

Part Used	Extracts	Experimental Approach	Key Results	References
**Aerial part**	Flavonic extract (Butanolic fraction)	Carrageenan-induced inflammation	Reduction in paw oedema volume by 8.55% at a concentration of 0.1 g/mL 27.15% at a concentration of 0.2 g/mL 33.01% at a concentration of 0.3 g/mL	[57]
**Aerial part**	Methanol extract	Carrageenan-induced inflammation	Inhibition by 56.2%	[171]
**Leaves**	Methanol extract	Carrageenan-induced inflammation. and Determination of C-reactive protein	Reduction in paw oedema volume by 40% A significant decrease in the inflammatory cells and C-reactive protein (CRP) level in serum samples	[40]
**Leaves and flowers**	Methanol extract	Cyclooxygenase inhibition	Flower: Cox-1 IC_50_ = 0.122 Cox-1 inhibition by 61.05% at a concentration of 0.033 mg/mL Leaves: Cox-1 inhibition by 5.33% at a concentration of 0.033 mg/mL	[35]
**Leaves**	Ethanol extract	Nitric oxide (NO) production	Decreases the cellular nitric oxide (NO) generation by 29% at 200 mg/mL Decreases the ulcer index (UI) from 2.66 mm to 1.66 mm	[133]
**Leaves**	Methanol extract	Lipoprotein peroxidation assay	Reduction in VLDL-LDL peroxidation up to 47%	[169]
**Leaves**	Methanol extract	Cyclooxygenase inhibition (TMPD and PGE2 assays)	COX-1 inhibition by 51.3% in TMPD assay COX-1 inhibition by 40.6% in PGE2 assay	[125]
**Leaves**	Aqueous extract	Cyclooxygenase inhibition and proinflammatory markers expression	Decrease in the COX-2 expression and EC-LPS effect on colon proinflammatory proteins	[170]
**Leaves**	Methanol extract	5-lipoxygenase assay and nitric oxide (NO) production	Inhibition of 5-lipoxygenase: IC_50_ = 79 ± 0.8 mg/L NO production by 66% at 600 mg/L	[56]
**Aerial part**	Aqueous extract	Dosage of tumor necrosis factor (TNF-α) and interleukin-6 (IL-6)	Decrease in lipid peroxidation and pro-inflammatory cytokines (IL-6 and TNF-α) levels	[169]

### 6.5. Antiproliferative and Cytotoxic Activities

Cancer has historically been considered one of the most severe human diseases, primarily due to its elevated rates of morbidity and mortality [176]. Conventional cancer treatments such as radiotherapy, surgery, and chemotherapy have, to some extent, saved lives; however, their effectiveness is hindered by severe side effects and high rates of relapse, limiting their ability to control and, in certain cases, cure tumors. Consequently, there is an urgent need for the development of more diverse and effective medications from various sources, as emphasized by Jemal et al. [176]. Natural compounds derived from plants are gaining attention for their lower toxicity and high selectivity toward targets compared to synthetic chemotherapeutic drugs. Therefore, exploring the potential of medicinal plants as anticancer agents is crucial. *G. alypum* has exhibited notable anticancer potential, as evidenced by multiple studies demonstrating potent effects of various extracts against tumor cell lines. Nouir et al. investigated the antiproliferative effects of *G. alypum* leaf extracts on SW620 at various concentrations, revealing concentration-dependent inhibition of cell line proliferation. The methanolic extract, obtained through sonication, demonstrated the highest antiproliferative effect, with an IC_50_ value of 20 µg/mL after 48 h of incubation [148]. Likewise, Friscic et al. evaluated the anticancer potential of methanolic leaves extracts from *G. alypum*, against the A1235 glioblastoma cell line using the MTT assay, resulting in a promising IC_50_ value of 231.43 µg/mL [125].

### 6.6. Other Activities

The process of wound healing presents four sequential phases: homeostasis, inflammation, proliferation, and remodeling. These stages involve intricate biological and chemical mechanisms that safeguard the wounded area against infections while fostering the regeneration of tissues. *G. alypum*, a plant with a history of use in traditional medicine for addressing surface wounds, underwent testing to evaluate its effectiveness in promoting wound healing.

The wound healing activity of *G. alypum* extract was investigated by Ghlissi et al. and they examined the burn wound healing activity of the methanolic extract of *G. alypum*, (GAME) on a group of 20 rats with burn induction during sixteen days of treatment. As a result, the macroscopic characteristics and size variations of burn wounds in rats treated with GAME (group specified) and other groups show that on day 4, the untreated burn-injured animals exhibited extensive areas of unhealed wounds. By day 16, these wounds were covered with irregularly dried burn eschar, displaying signs of wound contraction. In contrast, rats treated with GAME consistently demonstrated enhanced wound coverage by day 4, and this improvement persisted through day 16. Notably, the wound area significantly decreased in the GAME-treated group on day 12 and day 16 when compared to the untreated group. The visual observations align with variations in hydroxyproline content, as indicated in Table 6. The group treated with GAME exhibited an increased level of hydroxyproline (26.23 ± 1.90 mg/g of tissue), which suggests a more accelerated wound healing process compared to the untreated group. In the healed biopsies of the CC-treated group, significant tissue improvement was noted, with structured epidermal layers but persistent signs of inflammation. However, in the tissue sections of the GAME-treated group, the skin layers were clearly identified, with normal epidermis and dermis [40].

Ghlissi et al. evaluated the anticoagulant effect of the sulphated polysaccharide in vivo and in vitro, extracted from *G. alypum* leaves (GASP). The in vitro anticoagulant activity of GASP was performed by the activated partial thromboplastin time (aPTT), thrombin time (TT), and prothrombine time (PT) assays, and the in vivo anticoagulant activity of GASP was tested on male Wistar rats injected subcutaneously with 200 mg/kg b.w and 500 mg/kg b.w of GASP. Both doses of GASP demonstrate effective anticoagulant activities both in vitro and in vivo. Furthermore, toxicological assessments involving plasma transaminases, oxidative status, and liver tissue histopathology confirm the absence of hepatocyte necrosis, oxidative stress, or morphological alterations in the liver. Notably, GASP administered at 500 mg/kg body weight emerges as a more promising candidate for further evaluation, given its superior pharmacological and toxicological profile compared to doses of 200 mg/kg body weight and calciparin [132].

The nephroprotective effect was investigated using the methanolic extract derived from *G. alypum* leaves on adult male Wistar rats. Feriani et al. demonstrated the potential of *the G. alypum* methanolic extracts to mitigate the nephrotoxic effects induced by Deltamethrin in rats. *G. alypum* administration effectively restored disrupted parameters such as creatinine, urea, and uric acid levels caused by Deltamethrin treatment. Furthermore, *G. alypum* administration led to a reduction in kidney lipid peroxidation and protein carbonyl content, both of which were elevated by Deltamethrin exposure. These biochemical findings were supported by *G. alypum* ability to protect against DNA damage and its positive impact on kidney fibrosis, as observed in histopathological studies following deltamethrin administration. These outcomes align with the phytochemical composition of this plant, suggesting that *G. alypum* leaves may serve as a valuable source of antioxidants, contributing to potential health benefits [131].

In another study, Chokri and collaborators evaluated the myorelaxant and spasmolytic effects of the methanolic extract of *G. alypum* on rabbit jejunum. Methanolic extract has a relaxant and spasmolytic effect on rabbit jejunum, thus confirming the use of this plant on traditional medicine for the treatment of gastrointestinal disorders [61].

### 6.7. Toxicology and Safety

Ghlissi et al. evaluated the toxicity of *G. alypum* by inspection of plasma transaminases and the status of liver tissue of the sulfated polysaccharide extract from *G. alypum* leaves. Such a work confirmed that such compounds are not responsible for the negative changes in the liver [132]. The in vivo effect of the aqueous extract of *G. alypum* leaves was investigated to evaluate the acute toxicity in male Wistar rats based on their body weight at different concentrations (0, 2.5, 5, 6, 7, 8, 9, 10, 12, and 14 g/kg). The LD_50_ value of the GA aqueous extract was 14.5 g/kg, appearing to have minimal detrimental effects in terms of LD_50_ values [157]. Moreover, Skim et al. investigated the acute and chronic toxicity of water extract of *G. alypum* leaves from Morocco on Wistar rats. For the evaluation of the acute toxicity, six different concentrations of the extract (1, 2, 4, 6, 8, and 10 g/kg bw) were administered orally to the rats, and for the chronic toxicity, the rat group was given, orally, 0.7 g/kg bw of the plant infusion daily for 8 weeks. All rats treated with different concentrations of the infusions of *G. alypum* remained alive during the 7 days of observation. This suggested that the LD_50_ of the infusion extract was higher than 10 g/kg. The infusion of the leaves of *G. alypum* at a dose of 0.7 g/kg exerted no ill effects during the treatment for 8 weeks [54]. In the study by Shanab and Auzi based on Lorke’s method of aqueous extract of Libyan *G. alypum* in albino mice, injected with different concentration of the extract viz., 10 mg/kg, 100 mg/kg, and 1000 mg/kg and 1600, 2900, and 5000 mg/kg body weight, no mortality was found [177]. In another study conducted by Fehri et al., it was demonstrated that even at a dose of 10,000 mg/kg, the extract does not cause death when supplied orally. The extract’s LD_50_ was reported to be 2750 and 2550 mg/kg when administered intraperitoneally to female and male mice, respectively [63].

These studies suggest that the different portions of the plant did not exhibit major side effects when compared to the doses examined, and further chronic toxicity studies are required to investigate the adverse consequences of *G. alypum* use for an extended period.

## 7. Conclusions

In this review, comprehensive information on the ethnobotanical uses, phytochemistry, pharmacology, and toxicological studies of *Globularia alypum* L. is summarized. This plant, abundant in the Mediterranean region, is employed by diverse populations for treating various ailments. Ethnobotanical surveys have highlighted a broad spectrum of applications influenced by the plant part used, the treated ailment, mode of use, and geographical region. The intimate relationship each population shares with the plant is evident, with knowledge transmission in traditional medicine forming the foundation for regional variations in its utilization.

The observed disparities in biological outcomes among regions can be attributed to variations in bioactive compounds expressed by *G. alypum*. Distinct environmental conditions affecting the plant’s chemical composition explain its diverse effects in different regions. Phytochemical analyses have identified a range of bioactive compounds, such as flavonoids, phenolic acids, lipids, alkanes, alcohols, and terpenoids. The chemical profile of *G. alypum* varies based on collection location, likely influenced by climatic factors affecting secondary metabolite synthesis and secretion through epigenetic control.

Promising results regarding antioxidant and anti-hyperglycemic effects have been reported. Studies indicate that phenolic compounds in *G. alypum* extracts play a crucial role, exhibiting notable antioxidant and antidiabetic actions. This underscores the significance of pharmacological research in uncovering novel natural molecules from this plant. *G. alypum* extracts also exhibit other biological activities, including anticancer and anti-inflammatory effects. While the plant shows efficacy against these pathologies, the precise pharmacodynamic and pharmacokinetic mechanisms remain unclear.

It should be noted that the present work is a narrative compilation summarizing the available knowledge rather than a systematic review. We therefore emphasize the need for future systematic investigations focusing on standardized extract characterization, study quality assessment, and structure–activity relationship analyses. Furthermore, comprehensive preclinical and clinical research remains essential to elucidate the mechanisms of action of *G. alypum* bioactive compounds, validate its pharmacological potential in clinical settings, and assess long-term safety to support its development as a standardized phytotherapeutic agent.

## Figures and Tables

**Table 1:** Ethnomedicinal uses of *G. alypum*.

Region	Part Used	Mode of Preparation	Traditional Use	References
**Tafilalet (Southeast Morocco)**	Not reported	Not reported	Diabetes	[27]
**Regions of Fez-Boulemane, Meknes-Tafilalet, and Marrakech-Tensift-Al Haouz (Morocco)**	Leaves	Decoction	Skin cancer	[92]
**Guercif province (Northeastern Morocco)**	Leaves	Decoction	Diabetes	[81]
**Tata province (Morocco)**	Leaves	Decoction, infusion	Hypoglycemic, digestive, choleretic, laxative, purgative, stimulant, depurative, antiseptic, antimycotic, constipation, gastric ulcer, abscesses, cutaneous neoplasms	[78]
**El Jadida city (Morocco)**	Leaves	Decoction	Appetite stimulant, bladder ailment, burns healing, diabetes, skin boutons, tuberculosis, wounds	[93]
**Ksar lakbir district (Northwest of Morocco)**	Leaves	Decoction	Hypoglycemic, digestive, helminthiasis and bilious, stimulation	[91]
**Taza province (Northern Morocco)**	Leaves	Decoction, powder	Stomachic, purgative, carminative, anti-rheum, anxiolytic, antidiabetic, menstrual pain, analgesic	[90]
**Agadir Ida Ou Tanane Province (Southwest Morocco)**	Leaves, steam, whole plant	Infusion, decoction, powder, friction	respiratory, digestive, dermatological, circulatory, neurological, and urinary disorders, and diabetes	[80,94]
**Fez-Boulmane region (Morocco)**	Leaves	Decoction	Diabetes	[8]
**Djebel Zdimm (Setif, East of Algeria)**	Leaves, seeds	Nd	Anti-jaundice, anti-anemic, and anti-nervousness activities	[86]
**Oriental region (Morocco)**	Leaves, whole plant	Decoction, infusion, powder	Diabetes, digestive system pathologies, allergy	[83]
**Chefchaouen province (Morocco)**	Leaves	Infusion	Diabetes	[95]
**Northeastern Morocco**	Leaves	Decoction	Kidney stones, pyelonephritis	[82]
**Agadir (Southwest of Morocco)**	Not reported	Not reported	Intestine disorders, burns and wounds healing	[84]
**Tizi n’ Test (Taroudant province, Morocco)**	**Leaves**	Decoction	Diabetes	[28]
**Bordj Bou Arreridj (Northeast Algeria)**	Inflorescences	Infusion, decoction	Depurative, diuretic, laxative, purgative, stomachic, and diaphoretic	[36]
**Beni Mellal province (Morocco)**	Leaves	Decoction, infusion	Diabetes	[79]
**Northeastern Algeria**	Leaves	Decoction	Diabetes	[85]
**Djebel Messaad region (M’Sila, Algeria)**	Leaves	Infusion, powder	Stomach problems, diarrhea, menstrual pain, eczema, burns and wounds	[88]
**Bissa region (Northeastern, Algeria)**	Leaves	Decoction	Diabetes, influenza, gases, jaundice	[77]
**Tessala region (Algeria)**	Not reported	Infusion	Constipation	[96]
**Semi-arid region of Algeria**	Not reported	Not reported	Constipation, diarrhea, gastritis	[97]
**Hodna region (Algeria)**	Aerial parts, whole plant	Decoction	Hypertension, eczema	[87]
**Batna region (East of Algeria)**	Leaves, flowers	Decoction	Digestive disorders	[89]
**Eastern region of Libya**	Not reported	Not reported	Diuretic, gastritis, hypertension, abortion miscarriage, metritis, ovary stimulant, stroke, vaginal diseases, diarrhea, ulcer, colic, eczema, psoriasis, dermatitis, vaginitis, hemostatic, premenstrual syndrome, delayed menses	[98]
**Oulad Daoud Zkhanine (Nador province, Northeastern Morocco)**	Leaves, fruits	Decoction, infusion	Vomiting, constipation, antidiabetic, eczema, wounds and injuries	[99]
**Central Russikada (Northeastern Algeria)**	Not reported	Not reported	Constipation	[100]
**Middle Oum Rbia (Morocco)**	Leaves	Not reported	Digestives disorders	[101]
**Tangier, Tetouan, and Chefchaouen cities (Northern Morocco)**	Leaves	Decoction, infusion	Diabetes	[102]
**Kantara (Algeria)**	Aerial parts	Infusion	Headache, antipyretic, stomach-ache, poisoning, indigestion, gallbladder pains	[103]
**Central High Atlas (Morocco)**	Flower	Infusion	Anti-ulcer	[104]
**Oriental region (Morocco)**	Leaves	Decoction	Antidiabetic, laxative, cholagogue, stomachic, sudorific, purgative	[21]
**Constantine and Mila (Northeastern Algeria)**	Not reported	Not reported	Gynecological diseases, blood purification, antiseptic, excitant, antifungal, wounds, respiratory system diseases	[105]
**Tassili N’ajjer (Central Sahara, Algeria)**	Aerial parts	Decoction	Constipation, diabetes, fever, mycosis	[106]
**M’Sila (North Algeria)**	Flowered tops	Infusion, decoction	Antidiabetic, digestive disorders, leishmanicidal, eczema	[17]
**Eastern Mallorca (Balearic Islands)**	Leaves	Infusion	Antihypertensive	[107]
**Sefrou (Middle Atlas, Morocco**	Flower	Decoction	Diabetes	[25]
**Tlemcen National Park (Northwest Algeria)**	Leaves	Infusion	Menstrual cramp, diabetes	[108]
**Tiaret (Algeria)**	Not reported	Not reported	Diabetes, colonic disorders, parasitic infections, and stomachic disorders	[109]
**Ouargla province (Southeastern Algeria)**	Leaves	Decoction, cataplasme	Diabetes	[18]
**Chlef (Algeria)**	Leaves	Decoction	Flatulence	[110]
**Rural municipalities of Haizer and El Asnam (Bouira province, northern Algeria)**	Leaves	Infusion, decoction	Diabetes, dizziness, colic	[111]
**Bechar district (Southwest Algeria)**	Leaves, flowers	Infusion	Urinary incontinence	[112]
**Tiaret Mountains (Western Algeria)**	Not reported	Not reported	Antiparasitic, stomachic, diabetes and colon	[113]
**El Oued region (Southeastern Algeria)**	Leaves, flowers	Decoction	Antifungal, stomach cramp	[114]
**Djelfa (Central Steppe region, Algeria)**	Leaves, aerial parts	Decoction, maceration, powder, compress	Joint pain, influenza, diabetes, gales, injuries, constipation, hypertension	[115]
**Bigoudine watershed (Western High Atlas, Moroccan)**	Leaves	Decoction	Hypoglycemics, anti-rheumatics, stomachics, purgatives, and laxatives	[116]
**Aures (Algeria)**	Whole plant	Infusion	Fever, stomach-ache, weakness	[117]
**Ouled Dabbeb (Southern Tunisia)**	Leaves	Decoction	Furunculosis, urinary incontinence	[118]
**High Atlas (Morocco)**	Leaves	Infusion	General health, pediatric	[119]
**Khemisset (Morocco)**	Leaves	Infusion	Diabetes	[120]
**Coastal region of Libya**	Shoot	Decoction, infusion	Gastritis	[121]
**Eastern Morocco**	Leaves	Decoction	Digestive problems	[122]
**Etna Regional Park (Eastern Sicily, Italy)**	Leaves, aerial parts	Decoction	Renal stones	[123]
**Algerian steppe**	Whole plant	Paste	Wounds and fractures for animals	[124]
**Meknes-Tafilalet region (Morocco)**	Leaves	Decoction	Diabetes	[23]

**Table 4:** Antioxidant results of *G. alypum* extracts.

Part Used	Extracts	Used Methods	Key Results	References
Leaves	EtOAc	DPPH	IC_50_ = 12.3 ± 3.83 µg/mL	[3]
		ABTS	IC_50_ = 37.0 ± 2.45 µg/mL	
		FRAP	531.1 ± 1.08 mgAAE/g DW	
	Chloroform	DPPH	IC_50_ = 69.8 ± 1.89	
		ABTS	IC_50_ = 114.6 ± 0.63 µg/mL	
		FRAP	473.2 ± 1.88 mgAAE/g DW	
Aerial part	Methanol–water	DPPH	EC_50_ = 08.77 ± 0.04 mg/L	[6]
Leaves	Ethanol	DPPH	IC_50_ = 29.8 ± 0.20 µg/mL	[139]
		β-carotene	IC_50_ = 42.53 ± 0.24 µg/mL	
		ABTS	89.5 ± 0.32 mg EVC/g DW	
Leaves	Methanol–water	DPPH	IC_50_ = 39.30 ± 1.50 µg/mL	[1]
		β-carotene	54.20 ± 3.47%	
Leaves	Ethanol–water	DPPH	IC_50_ = 6.40 ± 0.19 µg/mL	[20]
		ABTS	3.02 ± 0.09 TEAC	
Flowers	Methanol	DPPH	IC_50_ = 0.004 ± 0.001 mg/mL	[35]
		β-carotene	IC_50_ = 0.26 ± 0.02 mg/mL	
Leaves	Methanol	DPPH	IC_50_ = 0.031 ± 0.004 mg/mL	
		β-carotene	IC_50_ = 0.51 ± 0.03 mg/mL	
Flowers	Methanol–water	FRAP	24.27 ± 1.57 mmol Fe^2+^/L	[14]
		DPPH	230 ± 7.26 µmol TEAC g^−1^	
		FRAP	21.81 ± 1.4 mmol Fe^2+^/L	
Aerial parts	Diethyl ether	DPPH	IC_50_ = 20.54 ± 0.48 µg/mL	[42]
		FRAP	EC_50_ = 283.7 ± 82.59 µg/mL	
Leaves	Ethanol	DPPH	IC_50_ = 4.95 ± 0.94 mg/mL	[44]
Leaves	Petroleum ether	DPPH	IC_50_ = 285.22 mg/mL	[53]
	Dichloromethane		IC_50_ = 512.06 mg/mL	
	Acetone		IC_50_ = 20.33 mg/mL	
	Water		IC_50_ = 15.58 mg/mL	
	Methanol–water		IC_50_ = 512.06 mg/mL	
Leaves	Ethanol–water	ABTS	0.172 mmol TE/g DW	[6]
Leaves	Methanol	DPPH	IC_50_ = 112.32 ± 1.42 µg/mL	[7]
		ABTS	IC_50_ = 18.65 ± 1.21 µg/mL	
Leaves	Methanol	DPPH	IC_50_ = 25.65 µg/mL	[144]
	EtOAc		IC5_50_ = 48.28 µg/mL	
Steams	Methanol	DPPH	IC_50_ = 22.11 µg/mL	
	EtOAc		IC_50_ = 33.67 µg/mL	
Roots	Methanol	DPPH	IC_50_ = 10.01 µg/mL	
	EtOAc		IC_50_ = 18.85 µg/mL	
Leaves	Water	FRAP	8.9 ± 0.006 mM TE	[19]
	Methanol		8.5 ± 0.004 mM TE	
	EtOAc		5.8 ± 0.005 mM TE	
	Petroleum ether		1.1 ± 0.005 mM TE	
	Water	Reducing power	4.5 ± 0.006 mM TE	
	Methanol		4.2 ± 0.004 mM TE	
	EtOAc		3.2 ± 0.005 mM TE	
	Petroleum ether		1.3 ± 0.005 mM TE	
Leaves	Ethanol–water	DPPH	IC_50_ = 8.31 ± 0.2 µg/mL	[20]
		ABTS	3.02 ± 0.09 TE	
Leaves	Methanol	DPPH	IC_50_ = 203 ± 1.83 µg/mL	[131]
		ABTS	IC_50_ = 168.85 ± 2.05 µg/mL	
		Reducing power	EC_50_ = 15.25 ± 0.49 mg/mL	
Aerial parts	Methanol	DPPH	IC_50_ = 17.25 µg/mL	[125]
Leaves	Aqueous	DPPH	IC_50_ = 60.24 ± 0.33 µg/mL	[41]
	EtOAc		IC_50_ = 10.58 ± 0.08 µg/mL	
	Butanol		IC_50_ = 10.54 ± 0.02 µg/mL	
Leaves	Ethanol	DPPH	IC_50_ = 15.00 ± 1.22 µg/mL	[136]
		TAC	26.23 ± 1.65 (GAE/g DW)	
Whole plant	Methanol–water	DPPH	88.34%	[5]
Aerial part	Decoction	DPPH	IC_50_ = 25.62 ± 0.48 µg/mL	[145]
	Aqueous	FRAP	EC_50_ = 0.33 ± 0.01 mg/mL	
	Infusion	DPPH	IC_50_ = 23.04 ± 0.73 µg/mL	
	Aqueous	FRAP	EC_50_ = 0.31 ± 0.01 mg/mL	
	Maceration	DPPH	IC_50_ = 85.37 ± 1.48 µg/mL	
	Aqueous	FRAP	EC_50_ = 0.68 ± 0.19 mg/mL	
Leaves	Diethyl ether	DPPH	84.1 ± 3.05%	[146]
Leaves	Methanol	DPPH	IC_50_ = 16.1 ± 1.1 µg/mL	[147]
		ABTS	IC_50_ = 49.5 ± 2.5 µg/mL	
		β-carotene	IC_50_ = 288.6 ± 7.2 µg/mL	
		FRAP	IC_50_ = 50.1 ± 4.5 µg/mL	
Leaves	Soxhlet	ABTS	IC_50_ = 0.25 ± 0.01 mg/mL	[148]
	Methanol	β-carotene	IC_50_ = 0.65 ± 0.05 mg/mL	
	Sonication	ABTS	IC_50_ = 0.18 ± 0.00 mg/mL	
	Methanol	β-carotene	IC_50_ = 0.28 ± 0.09 mg/mL	
	Maceration	ABTS	IC_50_ = 0.04 ± 0.01 mg/mL	
	Methanol	β-carotene	IC_50_ = 0.15 ± 0.00 mg/mL	

**Table 5:** Antimicrobial results of *G. alypum* extracts.

Used Part	Extracts	Tested Strains	Key Results	References
Leaves	Methanol–water	*Bacillus subtilis*	Φ = 9.0 mm	[1]
		*Staphylococcus aureus*	Φ = 8.0 mm	
		*Pseudomonas aeruginosa*	No inhibition	
		*Escherichia coli*	No inhibition	
		*Candida. albicans*	Φ = 11.0 mm	
Leaves	Methanol	*Listeria monocytogenes*	Φ = 15.0 ± 0.3 mm	[40]
		*Staphylococcus aureus*	Φ = 16.0 ± 0.2 mm	
		*Micrococcus luteus*	Φ = 16.0 ± 0.1 mm	
		*Escherichia coli*	Φ = 19.0 ± 0.1 mm	
		*Salmonella enterica*	Φ = 20.0 ± 0.1 mm	
		*Klebsiella pneumoniae*	Φ = 18.0 ± 0.1 mm	
Leaves and flowers	Aqueous	*Staphylococcus aureus*	Infused: Φ = 15.0 mm	[151]
			Decocted: Φ = 10.0 mm	
			Percolated: Φ = 14.0 mm	
			Macerated: Φ = 12.0 mm	
		*Escherichia coli*	Infused: Φ = 13.0 mm	
			Decocted: Φ = 10.0 mm	
			Percolated: Φ = 10.0 mm	
			Macerated: Φ = 12.0 mm	
		*Pseudomonas aeruginosa*	Infused: Φ = 7.0 mm	
			Decocted: Φ = 13.0 mm	
			Percolated: Φ = 10.0 mm	
			Macerated: Φ = 10.0 mm	
		*Proteus Mirabilis*	Infused: Φ = 12.0 mm	
			Decocted: Φ = 10.5 mm	
			Percolated: Φ = 10.0 mm	
			Macerated: Φ = 10.5 mm	
Leaves	Methanol	*S. aureus ATCC 29213*	Metoh: Φ = 6.67 ± 0.33 mm	[144]
	Ethyl acetate		MIC = 2 mg/mL	
			MBC = 2 mg/mL	
			EtOAc: Φ = 07.0 ± 0.00 mm	
			MIC = 4 mg/mL	
			MBC = 4 mg/mL	
Stems	Methanol	*B. subtilis ATCC 3366*	Metoh: Φ = 8.33 ± 0.67 mm	
	Ethyl acetate		MIC = 16 mg/mL	
			MBC = 16 mg/mL	
			EtOAc: Φ = 10.83 ± 0.17 mm	
			MIC = 2 mg/mL	
			MBC = 2 mg/mL	
Roots	Methanol	*Micrococcus luteus*	Metoh: Φ = 9.00 ± 0.00 mm	
	Ethyl acetate		EtOAc: Φ = 12.17 ± 0.17 mm	
Leaves	Methanol	*P. aeruginosa ATCC 27853*	Metoh: Φ = No inhibition	
Steams	Ethyl acetate		EtOAc: Φ = No inhibition	
Roots				
Leaves	Methanol	*E.coli ATCC 25922*	Metoh: Φ = No inhibition	
Steams	Ethyl acetate		EtOAc: Φ = No inhibition	
Roots				
Leaves	Methanol	*C.albicans ATCC 10231*	Metoh: Φ = No inhibition	
Steams	Ethyl acetate		EtOAc: Φ = No inhibition	
Aerial parts	E.O	*S. typhimurium ATCC 13311*	B: Φ = 33 mm	[55]
			K: Φ = 36 mm	
		*K. pneumoniae ATCC 700603*	B: Φ = 14 mm	
			K: Φ = 11 mm	
		*P. mirabilis ATCC 35659*	B: Φ = 15 mm	
			K: Φ = No inhibition	
		*P. aeruginosa ATCC 27853*	B: Φ = 25 mm	
			K: Φ = 36 mm	
		*B. cereus ATCC 10876*	B: Φ = 11 mm	
			K: Φ = 12 mm	
		*B. subtilus ATCC 663313*	B: Φ = 20 mm	
			K: Φ = 22 mm	
		*E. faecalis ATCC 49452*	B: Φ = 23 mm	
			k: Φ = 25 mm	
		*L. monocytogenes ATCC 15313*	B: Φ = 24 mm	
			K: Φ = 40 mm	
		*S. aureus ATCC 25923*	B: Φ = 8 mm	
			K: Φ = No inhibition	
		*A. baumanii ATCC19606*	B: Φ = 21 mm	
			K: Φ = 22 mm	
		*C. freundii ATCC 8090*	B: Φ = 50 mm	
			k: Φ = 18 mm	
		*E. coli ATCC 25922*	B: Φ = 42 mm	
			K: Φ = 40 mm	
Leaves	Aqueous	*Staphylococcus aureus*	Aqueous: Φ = 12.5 ± 0.5 mm	[41]
	Butanol		Butanol: Φ = 15.33 ± 0.76 mm	
	EtOAc		EtOAc: Φ = 18 ± 0.46 mm	
		*Enterococcus faecalis*	Aqueous: Φ = 9.66 ± 0.5 mm	
			Butanol: Φ = 11.5 ± 0.35 mm	
			EtOAc: Φ = 13.5 ± 0.91 mm	
		*Pseudomonas aeruginosa*	Aqueous: Φ = 10.66 ± 0.76 mm	
			Butanol: Φ = 12.16 ± 0.61 mm	
			EtOAc: Φ = 15.5 ± 0.42 mm	
		*Escherichia coli*	Aqueous: No inhibition	
			Butanol: Φ = 9.33 ± 0.23 mm	
			EtOAc: Φ = 13 ± 0.36 mm	
		*Salmonella typhi*	Aqueous: Φ = 10 ± 0.4 mm	
			Butanol: Φ = 10.66 ± 0.47 mm	
			EtOAc: Φ = 11.05 ± 0.3 mm	
		*Aspergillus flavus*	Aqueous: Inhibition = No inhibition	
			Butanol: Inhibition = 37.87%	
			EtOAc: Inhibition = 43.93%	
		*Penicillium* spp.	Aqueous: Inhibition = No inhibition	
			Butanol: Inhibition = 47.82%	
			EtOAc: Inhibition = 58.69%	
		*F. oxysporum*	Aqueous: Inhibition = 19.69%	
			Butanol: Inhibition = 51.38%	
			EtOAc: Inhibition = 66.66%	
Leaves	Ethanol	*E. coli*	Φ = 14 mm	[136]
			MIC = 6.25 mg/mL	
			MBC = 12.5 mg/mL	
		*S. aureus*	Φ = 14 mm	
			MIC = 6.25 mg/mL	
			MBC = 12.5 mg/mL	
Leaves	Methanol	*S. mutans ATCC 25175*	Φ = 13 mm	
			MIC = 2560 µg/mL	
			MBC = 10,240 µg/mL	
		*S. oralis ATCC 6249*	Φ = 17 mm	
			MIC = 5120 µg/mL	
			MBC = 10,240 µg/mL	
		*P. gingivalis ATCC 33277*	Φ = 16 mm	
			MIC = 5120 µg/mL	
			MBC = 20,480 µg/mL	
		*S. salivarius ATCC 13419*	Φ = 9 mm	
			MIC = 5120 µg/mL	
			MBC = 10,240 µg/mL	
		*S. thermophilusATCC 19258*	Φ = 9 mm	
			MIC = 5120 µg/mL	
			MBC = 10,240 µg/mL	
		*L. plantarum ATCC 8014*	Φ = 10 mm	
			MIC = 5120 µg/mL	
			MBC = 20,480 µg/mL	
		*E. feacalis ATCC 29212*	Φ = 12 mm	
			MIC = 10,240 µg/mL	
			MBC = 20,480 µg/mL	
		*S. aureus ATCC 25923*	Φ = 9 mm	
			MIC = 5120 µg/mL	
			MBC = 10,240 µg/mL	
		*M. luteus ATCC 10240*	Φ = 12 mm	
			MIC = 5120 µg/mL	
			MBC = 20,480 µg/mL	
		*E. coli ATCC 25922*	Φ = 10 mm	
			MIC = 10,240 µg/mL	
			MBC = 10,240 µg/mL	
		*C. albicans ATCC 90028*	Φ = 11 mm	
			MIC = 2560 µg/mL	
			MBC = 5120 µg/mL	
		*C.creusi ATCC 6258*	Φ = 7 mm	
			MIC = 10,240 µg/mL	
			MBC = 10,240 µg/mL	
		*A.brasiliensisATCC 16404*	Φ = 8 mm	
			MIC = 10,240 µg/mL	
			MBC ≥ 20,480 µg/mL	
		*A. sfumigatusATCC 204305*	Φ = 14 mm	
			MIC = 10,240 µg/mL	
			MBC ≥ 20,480 µg/mL	
		*C. neoformansATCC 14116*	Φ = 19 mm	
			MIC = 5120 µg/mL	
			MBC ≥ 20,480 µg/mL	
Whole plant	Methanol	*P.aeruginosa ATCC 27853*	Methanol: Φ = 15 mm	[150]
	Ethyl acetate		Chloroform: Φ = 9 mm	
	Chloroform		EtOAc: Φ = 16 mm	
	Aqueous		Aqueous: Φ = 10 mm	
		*E. coli ATCC 25922*	Methanol: Φ = No inhibition	
			Chloroform: Φ = 9 mm	
			EtOAc: Φ = 10 mm	
			Aqueous: Φ = No inhibition	
		*S.typhimurium ATCC 13311*	Methanol: Φ = 11 mm	
			Chloroform: Φ = 11 mm	
			EtOAc: Φ = 12 mm	
			Aqueous: Φ = 9 mm	
		*A. baumanii ATCC 19606*	Methanol: Φ = 9 mm	
			Chloroform: Φ = 12 mm	
			EtOAc: Φ = 9 mm	
			Aqueous: Φ = 11 mm	
		*C. freundii ATCC 8090*	Methanol: Φ = 9 mm	
			Chloroform: Φ = 8 mm	
			EtOAc: Φ = 8 mm	
			Aqueous: Φ = 9 mm	
		*P. mirabilis ATCC 35659*	Methanol: Φ = 9 mm	
			Chloroform: Φ = 8 mm	
			EtOAc: Φ = 8 mm	
			Aqueous: Φ = 9 mm	
		*K. pneumoniae ATCC 700603*	Methanol: Φ = No inhibition	
			Chloroform: Φ = No inhibition	
			EtOAc: Φ = No inhibition	
			Aqueous: Φ = 15 mm	
		*S.aureus ATCC 25923*	Methanol: Φ = 16 mm	
			Chloroform: Φ = 13 mm	
			EtOAc: Φ = 20 mm	
			Aqueous: Φ = 12 mm	
		*B. cereus ATCC 10876*	Methanol: Φ = 8 mm	
			Chloroform: Φ = 11 mm	
			EtOAc: Φ = 9 mm	
			Aqueous: Φ = No inhibition	
		*E.faecalis ATCC 49452*	Methanol: Φ = No inhibition	
			Chloroform: Φ = 9 mm	
			EtOAc: Φ = 10 mm	
			Aqueous: Φ = No inhibition	
		*L. monocytogenes ATCC 15313*	Methanol: Φ = No inhibition	
			Chloroform: Φ = 0.7 mm	
			EtOAc: Φ = 0.9 mm	
			Aqueous: Φ = No inhibition	
Aerial parts	Methanol	*B. cereus ATCC 11778*	Φ = 8.3 ± 0.6 mm	[125]
		*B. subtilis ATCC 6633*	Φ = 7.7 ± 0.6 mm	
		*E. faecalis ATCC 29212*	Φ = No inhibition	
		*S. aureus ATCC 6538*	Φ = 25 ± 0.0 mm	
		*E. coli ATCC 10536*	Φ = No inhibition	
		*K. pneumoniae MFBF 10402*	Φ = No inhibition	
		*P. aeruginosa ATCC 27853*	Φ = 9.7 ± 0.6 mm	
Leaves	Methanol	*S. mutans ATCC 25175*	Φ = 13 mm	[147]
			CMI = 2560 µg/mL	
			CMB = 10,240 µg/mL	
		*S. oralis ATCC 6249*	Φ = 17 mm	
			CMI = 5120 µg/mL	
			CMB = 10,240 µg/mL	
		*P. ingivalis ATCC 33277*	Φ = 16 mm	
			CMI = 5120 µg/mL	
			CMB = 20,480 µg/mL	
		*S. salivarius ATCC 13419*	Φ = 9 mm	
			CMI = 5120 µg/mL	
			CMB = 10,240 µg/mL	
		*S. thermophilusATCC 19258*	Φ = 17 mm	
			CMI = 2560 µg/mL	
			CMB = 10,240 µg/mL	
		*L. plantarum ATCC 8014*	Φ = 10 mm	
			CMI = 5120 µg/mL	
			CMB = 20,480 µg/mL	
		*E.feacalis ATCC 29212*	Φ = 12 mm	
			CMI = 10,240 µg/mL	
			CMB = 20,480 µg/mL	
		*S. aureus ATCC 25923*	Φ = 9 mm	
			CMI = 5120 µg/mL	
			CMB = 10,240 µg/mL	
		*M. luteus ATCC 10240*	Φ = 12 mm	
			CMI = 5120 µg/mL	
			CMB = 20,480 µg/mL	
		*E. coli ATCC 25922*	Φ = 8 mm	
			CMI = 10,240 µg/mL	
			CMB = 10,240 µg/mL	
		*C. albicans ATCC 90028*	Φ = 11 mm	
			MIC = 2560 µg/mL	
			MFC = 5120 µg/mL	
		*C. creusi ATCC 6258*	Φ = 7 mm	
			MIC = 10,240 µg/mL	
			MFC = 10,240 µg/mL	
		*A. brasiliensis ATCC 16404*	Φ = 8 mm	
			MIC = 10,240 µg/mL	
			MFC ≥ 20,480 µg/mL	
		*A. fumigatus ATCC 204305*	Φ = 14 mm	
			MIC = 10,240 µg/mL	
			MFC ≥ 20,480 µg/mL	
		*C. neoformans ATCC 14116*	Φ = 19 mm	
			MIC = 5120 µg/mL	
			MFC ≥ 20,480 µg/mL	

## Data Availability

No new data were created or analyzed in this study.
